# Anti-inflammatory, procollagen, and wound repair properties of topical insulin gel

**DOI:** 10.1590/1414-431X2023e12640

**Published:** 2023-05-15

**Authors:** P.P. Apolinário, F.C. Zanchetta, J.S.C. Breder, G. Adams, S.R. Consonni, R. Gillis, M.J.A. Saad, M.H.M. Lima

**Affiliations:** 1 Colégio Técnico de Campinas, Universidade Estadual de Campinas, Campinas, SP, Brasil; 2 Faculdade de Enfermagem, Universidade Estadual de Campinas, Campinas, SP, Brasil; 3 Faculty of Medicine and Health Science, University of Nottingham, Nottingham, UK; 4 Insituto de Biologia, Universidade Estadual de Campinas, Campinas, SP, Brasil; 5 Department of Service Sector Management, Sheffield Hallam University, Sheffield, UK; 6 Faculdade de Ciências Médicas, Universidade Estadual de Campinas, Campinas, SP, Brasil

**Keywords:** Diabetes mellitus, Healing, Insulin, Wounds

## Abstract

Diabetes mellitus is associated with impaired wound healing. The topical use of insulin is a promising therapy because it may favor all phases of the wound healing process. This study aimed to investigate the therapeutic outcomes of insulin gel in wounds of hyperglycemic mice. After diabetes induction, a 1-cm^2^ full-thickness wound was created on each animal's dorsum. The lesions were treated daily for 14 days with insulin gel (insulin group) or vehicle gel without insulin (vehicle group). Tissue samples were extracted on days 4, 7, 10, and 14 after the creation of the lesion. The samples were analyzed with hematoxylin/eosin and Sirius red staining, immunohistochemistry, Bio-Plex immunoassays, and western blotting. Insulin gel favored re-epithelialization at day 10 and increased the organization and deposition of collagen. Additionally, it modulated the expression of cytokines (interleukin (IL)-4 and IL-10) and increased the expression of arginase I, VEGF receptor 1, and VEGF on day 10. Activation of the insulin signaling pathway occurred via IRβ, IRS1, and IKK on day 10 and activation of Akt and IRS1 on day 14. These results suggested that insulin gel improved wound healing in hyperglycemic mice by modulating the expression of inflammatory factors, growth factors, and proteins of the insulin signaling pathway.

## Introduction

Diabetes mellitus (DM) is one of the major chronic non-communicable diseases (1) responsible for a heterogeneous set of metabolic disorders due to defects in insulin secretion and/or action, causing chronic hyperglycemia that leads to dysfunction and failure of different organs and wound healing (2). DM slows down the healing process, leading to a wound that does not heal. The healing process requires the collaboration between inflammatory cells and biochemical mediators that are stimulated by different factors. Altered cellular and biochemical mediators and activities are involved in the failure of wound healing in diabetics. Studies have shown that a chronic inflammatory state occurs in DM characterized by non-sequential release of pro- and anti-inflammatory cytokines, delayed angiogenic macrophage and neutrophil function, and impaired keratinocyte and fibroblast migration and proliferation, all of which result in an imbalance that leads to poor tissue repair (3).

Several studies have investigated new treatment strategies to improve wound healing in DM (4). Past research has shown that topical insulin plays an important role in wound healing (5). Insulin is a peptide hormone and growth factor that can reestablish damaged skin. In wound healing in experimental models, it has been linked to the regulation of oxidative and inflammatory responses, by decreasing the levels of reactive oxygen species (ROS), inducing the early recruitment of neutrophils, and accelerating the inflammatory response to injury by increasing the number of M2 macrophages and the rate of IL-10 clearance of dead tissue. Arginase 1, the precursor of nitric oxide, is upregulated in M2 macrophages exposed to interleukin (IL)-4 and IL-13, and its expression is associated with angiogenesis, re-epithelialization, and formation of granulation tissue (6). In addition, insulin facilitates macrophage chemotaxis and phagocytosis and the secretion of inflammatory mediators through the regulation of MCP-1 expression in injuries (7).

A previous study reported that insulin cream (0.50 U/100 g) reduces the wound healing time in hyperglycemic rats by improving the insulin signaling pathway and reducing the expression of the insulin receptors IRS1, IRS2, Shc, MAPK, and Akt. These changes provide strong evidence for the participation of topical insulin in cellular and molecular events in tissue reconstruction. Furthermore, the treatment also increased the expression of VEGF and stromal cell-derived factor 1α (SDF-1) in injured tissue (8). In second-degree burns of hyperglycemic rats, insulin cream increased inflammatory cell infiltration and collagen deposition compared with the vehicle group (9).

Moreover, a study to investigate the signal transduction pathways involved in insulin-induced monocytes/macrophages showed that insulin stimulates THP-1 cell (a monocyte/macrophage cell line) chemotaxis in a dose-dependent and insulin receptor-dependent manner. PI3K-Akt, SPAK/JNK, and p38 MAPK are key molecules in the insulin-induced signaling pathways that lead to the chemoattraction of THP-1 cells. Furthermore, both PI3K/Akt and SPAK/JNK signaling involve Rac1 activation, an important molecule in regulating cell motility. The major members of the MAPK family include Erk1/2, JNK, and p38 MAPK, among which Erk1/2 plays a key role in mediating the proliferation, differentiation, and migration of various cell types (10).

It should be acknowledged that there is great promise in translating the preclinical topical insulin treatment for wound healing research into the clinical setting (7). On the other hand, in clinical practice, an ideal moist environment must be created to enhance the wound healing rate. A gel-based dressing provides a moist environment and has good permeability, biocompatibility, and wettability, which favors healing (11).

This study aimed to analyze the molecular and cellular mechanisms of tissue repair in hyperglycemic mice with a wound submitted to topical treatment with insulin gel.

## Material and Methods

### Animals

Male isogenic 4-week-old C57BL/6J mice (18-20 g) purchased from the Breeding Centre of the State University of Campinas were used in this study (n=120). They were kept under pathogen-free standard conditions (room with a 12-h photoperiod, a temperature of 22±2°C, humidity of 60-80%, and food and water provided *ad libitum*). For inclusion in this study, the criterion for DM was a blood glucose level ≥250 mg/dL. Animals that did not develop DM or died prematurely were excluded, as were samples collected that were not usable for the different analyses. Animals that presented lethargy with mobility limitation or self-inflicted wounds were removed from the experiment and euthanized by anesthetic overdose followed by cervical dislocation. The animals were acclimatized to the laboratory conditions before the experiment began and were used only once. The sample size was calculated *a priori* using the G*Power 3.1.9.2 software. This calculation considered two groups at four time periods, according to the quantitative outcomes of repeated measures analysis of variance (ANOVA). A significance level of 5%, a test power of 80%, and an effect size of 0.25 were assumed. According to Cohen (12), this can be considered a medium effect size. The calculation resulted in a minimum sample of 82 mice (41 mice per group). To account for the exclusion criteria, a loss rate of 20% was considered. Thus, a minimum of 84 animals, with 42 per group, was required.

### Study design

**Figure 1:**
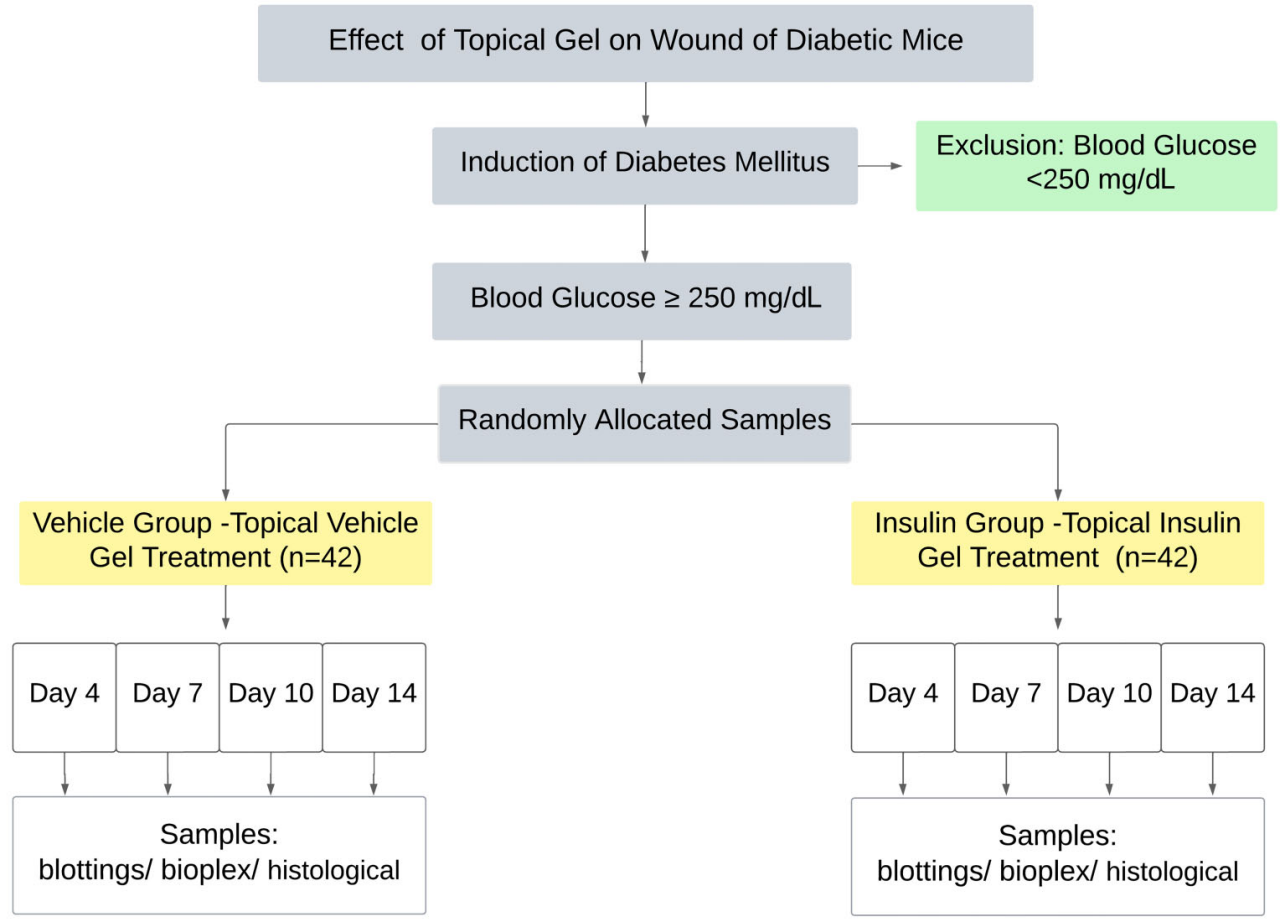
Study design.

Mice were injected with streptozotocin (Sigma, Inc., USA; 50 mg/kg per day × 5 days, intraperitoneal) dissolved in sodium citrate buffer 0.1 M (pH 4.5). This drug destroys pancreatic beta cells, resulting in a marked reduction in insulin release and, consequently, hyperglycemia. This model has been gradually accepted for type 1 DM research because of the high success rate and low spontaneous remission (13). Blood glucose was checked using an aliquot of blood obtained from the tail vein after 4 h of fasting (Accu-Chek Active, Roche, Germany) during the 14-day experiment. Mice presenting blood glucose ≥250 mg/dL were included in the study. The animals were monitored for weight and blood glucose after induction with streptozotocin. The capillary blood glucose (CBG) level of the animals was verified at the age of 9 weeks (DM confirmation date), 2 days after the injury incision, and at the date of excision of the injury. Body weight was confirmed at 6 weeks of age (first dose of streptozotocin), 9 weeks of age (at the time of the injury), and at the excision of the wound. The diabetic animals (n=89) were randomly allocated into two groups: the vehicle group (vehicle gel without insulin) (n=44) and the insulin group (n=45) (Figure 1). The wounds were assessed at four time points, namely days 4, 7, 10, and 14 after wounding, using six animals at each time point across the two groups.

### Experimental procedures

The animals were anesthetized intraperitoneally with 180 mg/kg ketamine (Ketalar; Parke-Davis, Brazil) and 8 mg/kg xylazine (National Pharmaceutical Chemistry Union S/A, Brazil) (14). A 1-cm^2^ plastic template and a surgical skin marker were used to delimit the lesion area at the dorsal midline. The animal's skin was removed with the aid of surgical scissors and forceps until it was at the level of the panniculus carnosus muscle. Treatment or vehicle (20 IU) was applied topically on the wound beds. The vehicle or treatment was placed on the injury immediately after the lesion (day 0) and reapplied daily until the end of the 14-day experiment. The wounds were not sutured or covered, they were allowed to heal by secondary intention, and there were no signs of infection. The wounds were observed every day for clinical signs of superficial infection such as purulent drainage, abnormal granulation tissue, abnormal foul odor, edema, induration, and erythema. Each animal was housed individually after wounding to prevent traumatic damage to the wounds by other mice. After the wounding procedure, animals received 20 mg/kg of intramuscular tramadol (Medley Pharmaceutical Industry, Brazil) for 2 days. This study was performed according to the internationally accepted principles for laboratory animal use and care, as found in the European Community guidelines (EEC Directive of 1986; 86/609/EEC) and the U.S. guidelines (NIH publication #85-23, revised in 1985) (15). All protocols followed the principles and guidelines adopted by the National Council for the Control of Animal Experimentation and were approved by the Ethical Committee for Animal Research (identification number 4234-1) of the State University of Campinas.

### Preparation of the insulin gel

To prepare the insulin gel, human crystalline regular insulin was incorporated into the vehicle at a concentration of 0.5 U/100 g (vehicle: hydroxypropyl methyl cellulose, low-molecular-weight chitosan, glycerol, and purified water) and stored at 2-8°C for all experiments.

### Macroscopic evaluation of the lesions

The wound area was measured immediately after wounding and at days 4, 7, 10, and 14 post-wounding using digital images obtained with a Canon PowerShot camera (SX400 IS 16MP × Optical zoom, Japan) and ImageJ^®^ software (National Institutes of Health, USA). The same examiner obtained all of the images. The percentage of the wound area was estimated as: [(total lesion area immediately after wounding at day 0 - wound area that was still not covered with epidermis) / lesion area at day 0] × 100.

### Histological examination of the wound tissue

The dissected skin samples were fixed by immersion in paraformaldehyde in distilled water (4%), dehydrated in increasing concentrations of ethanol, immersed in xylol, and embedded in paraffin. Histological sections (5 μm) were stained with hematoxylin and eosin (H&E) to evaluate wound morphology. Wound re-epithelialization was measured on days 1, 10, and 14 post-wounding via morphometric analysis of a wound section stained with H&E. The same examiner obtained all images using an optical microscope with a 1.2× objective lens (Zeiss Stemi SV6, Germany) with a Canon PowerShot A640 10MP (Japan) camera and captured the images using AxioVision software, version 4.8 (06-2009, Carl Zeiss, Germany). Re-epithelialization was calculated using the formula [(1 - {(distance traversed by epithelium) / (distance between wound edges)} × 100] for one median section per wound. The data are reported as the percentage of re-epithelialization at each time point. Statistical significance was determined using Student's *t*-test, with six animals per group at each time point.

### Histological analyses and index calculation

Tissue samples were evaluated for the following histological criteria: the extent of re-epithelialization, maturation, and organization of the epidermis, granulation tissue formation, collagenization, inflammatory cells, and scar formation in the dermis.

### Collagen fiber analyses

The Picrosirius red 1% technique (Sirius Red in a saturated solution of picric acid) under polarized light was used to evaluate type I and III collagen fibers (16). Fibers were identified by their birefringence pattern (type I: red, orange, and yellow; type III: green). Ten random fields (250× magnification) were visualized per group on extraction days 4, 7, 10, and 14 using an optical microscope (Leica^®^ DM4000B, Germany) with a LEICA^®^ DFC 280 camera connected to an AMD Athlon™ 6400 2.00 GHz (USA) computer equipped with an NVIDIA GeForce4 MX4000 (USA) video card and captured in the Image-Pro Plus (USA) 7.0 software, with 10× and 20× objective lenses.

### Immunohistochemistry

The tissue samples were embedded in paraffin and 5-μm sections were produced by a Zeiss^®^ microtome. The blades were kept in an oven at 37°C overnight and the tissue sections were deparaffinized using xylene. Rehydration was performed by immersion in decreasing concentrations of ethanol. Citrate buffer (10 mM, pH 6.0) was used to retrieve antigens in a pressure cooker for 40 min and a hydrophobic pen was used to circle tissue sections. Endogenous peroxidase was blocked by 3% H_2_O_2_ for 15 min at room temperature (RT). Before primary antibody incubation, tissue sections were incubated with blocking/antibody diluent (reagent A, Invitrogen^®^, USA) for 10 min at RT. The tissue sections were incubated with a primary antibody (arginase I [H-52: sc-20150, Santa Cruz Biotechnology, USA] or TNF-α [Ab 8348, Abcam, UK]) and kept at 4°C overnight in a staining tray in a humid chamber. The next morning, tissue sections were washed in 0.05 M phosphate-buffered saline (PBS) and then incubated with the appropriate secondary antibodies for 60 min at RT and in a moist chamber. Following a brief wash, tissue sections were incubated with chromogenic substrates (diaminobenzidine (DAB)) at RT. Counterstaining was performed using hematoxylin for 15 s followed by rinsing and bluing in flowing tap water for about 5 min. Then, tissue sections were dehydrated through increasing concentrations of ethanol and cleared in xylene. The slides were mounted with Entellan^®^ (Millipore, Germany) and dried in a chemical hood. A negative control was performed by omitting the primary antibody.

### Immunoassay for cytokines and chemokines in wound tissue

The levels of cytokines were determined using the Bio-Plex technique (Bio-Plex Pro Assays, Bio-Rad, USA). Tissue samples from the lesion area were collected on days 4 and 10 and immediately conditioned in nitrogen and kept in the freezer (-80°C). Subsequently, they were homogenized with PBS with complete Protease Inhibitor Cocktail (Roche), sonicated for 1 min and centrifuged at 177 *g* for 10 min at 4°C. The supernatant was collected and stored at -80°C. IL-1β, IL-4, IL-6, IL-10, TNF-α, and MCP-1 were evaluated by the Bio-Plex method using the Duo Set kit (R&D System, USA) and normalized by the protein concentration, as measured by the Bradford method.

### Western blotting

Tissue samples from days 4, 7, 10, and 14 post-wounding (four mice per time point) were used to measure protein levels by western blotting. The samples were homogenized in solubilization buffer (100 mM tris-hydroxymethyl-aminomethane pH 7.4, 10 mM sodium pyrophosphate, 100 mM sodium fluoride, 10 mM ethylenediaminetetraacetic acid, 10 mM sodium vanadate, 2 mM phenylmethylsulphonyl fluoride, and 0.1 mg/mL aprotinin) using a polytron PTA 20S generator (model PT 10/35, Brinkmann Instruments, USA) at maximum speed. The tissue extraction was performed using 10% volume Triton-X 100 and then centrifuged at 14,881 *g* at 4°C for 40 min. The supernatant protein concentration was quantified using the Bicinchoninic Acid Protein Assay Kit (Sigma-Aldrich) following the manufacturer's protocol and a microplate reader (FlexStation 3, Molecular Devices, USA) at 562 nm. The samples were mixed with Lammeli buffer containing 100 mM dithiothreitol and heated at 95°C for 5 min. Each sample (120 µg of protein) was subjected to gel electrophoresis in a Bio-Rad mini-gel apparatus (Mini-Protean SDS-Page, Bio-Rad). The electrotransfer of proteins from the gel to the nitrocellulose membranes was performed for 90 min at 120 V. Non-specific protein binding was reduced by incubating the membrane for 1 h at RT in a blocking buffer (5% bovine serum albumin [BSA], 10 mM Tris, 150 mM NaCl, and 0.02% Tween 20). Primary antibodies were diluted in 3% BSA, 10 mM Tris, 150 mM NaCl, and 0.02% Tween 20. Secondary antibodies were diluted in 1% BSA, 10 mM Tris, 150 mM NaCl, and 0.02% Tween 20. The antibodies used were: p44/42 MAPK (Erk1/2) (Cell 9102, Cell Signaling Technology, USA), VEGF (147) (sc-507, Santa Cruz Biotechnology), VEGF receptor 1 (Ab32152, Abcam), Akt1/2/3 (5C10) (sc-81434, Santa Cruz Biotechnology), IRβ (4b8) (Cell 3025, Cell Signaling Technology), IRS1 (Cell 2382, Cell Signaling Technology), phospho-SAPK/JNK (Thr183/Tyr185) (Cell 9251, Cell Signaling Technology), IKappa-βalpha (Cell 9242, Cell Signaling Technology), and arginase I (h-52) (sc-20150, Santa Cruz Biotechnology) at a dilution of 1:1000. The blots were analyzed by scanner densitometry (Image Lab 6.0, Bio-Rad). The results are reported as arbitrary units for diabetic animals.

### Statistical analysis

Student's *t*-test was used to compare groups. In some experiments, two-way ANOVA followed by the Bonferroni *post hoc* test was used. The significance was set at P<0.05.

## Results

### Skin wound healing and scar tissue

**Figure 2:**
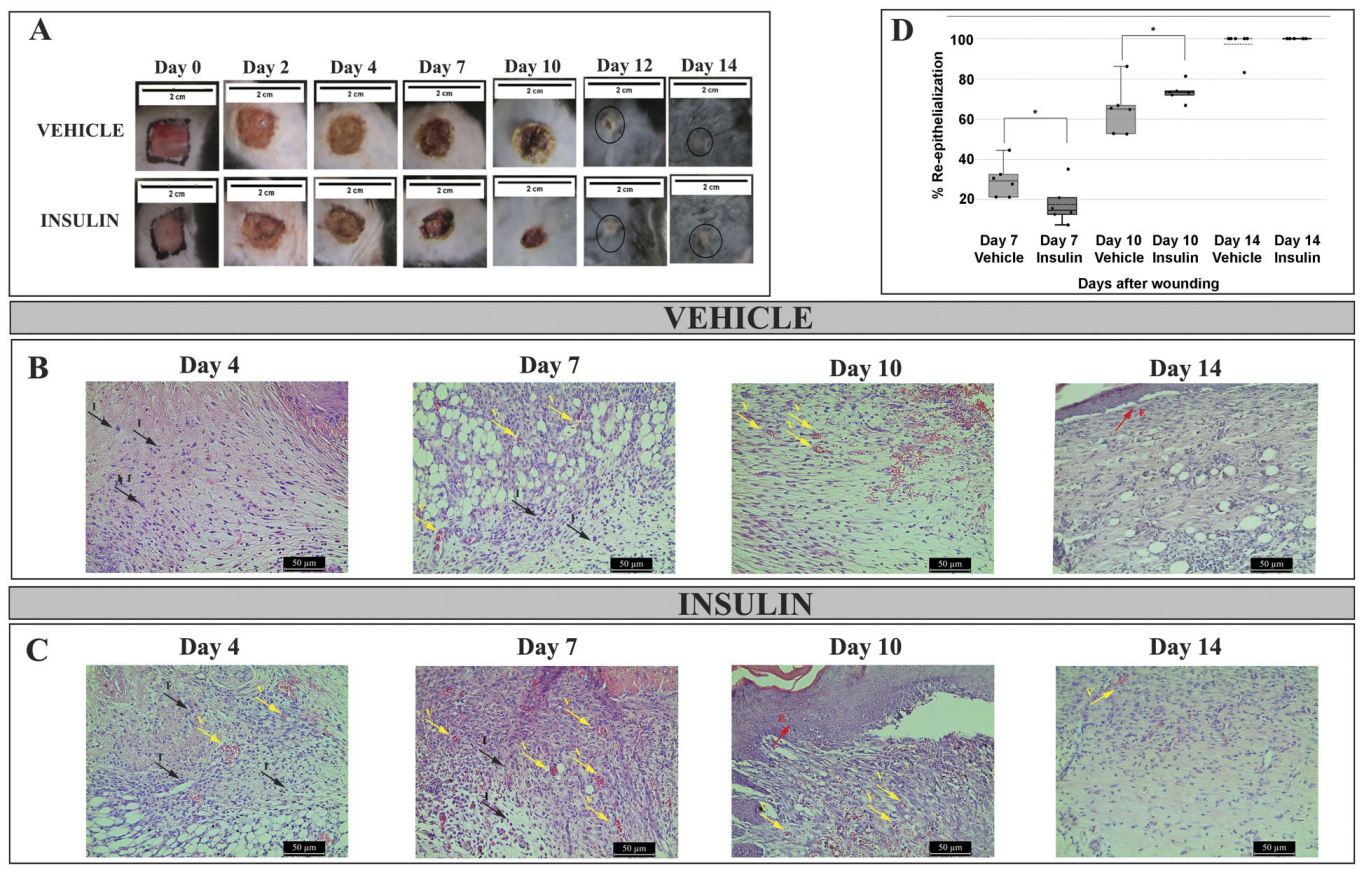
A, Representative macroscopic images of wounds of the vehicle and insulin groups at days 0, 2, 4, 7, 10, 12, and 14 after injury. Photomicrographs of sections of the central wound area stained with H&E from the vehicle group (**B**) and the insulin group (**C**) on days 4, 7, 10, and 14 days post-injury. Magnification: 20×; scale bar 50 μm. Arrows with ‘I’ (black) indicate inflammatory cells, arrows with ‘V’ (yellow) indicate new blood vessels, and arrows with ‘E’ (red) indicate the epidermis. **D**, Re-epithelialization percentage of the vehicle and insulin groups for days 7, 10, and 14 post-injury. The data are reported as medians and interquartile range (n=6 per group). *P<0.05, Student's *t*-test.

Observations of the general appearance of the lesions were made on days 0, 2, 4, 7, 10, 12, and 14. In the first days after the injury, the animals in the vehicle and insulin groups presented lesions with reddish tissue in the wound bed, a yellowish crust and edges at the beginning of contraction. At days 7 and 10, the lesions in both groups had a brown appearance, dry yellowish crusts, and contracted edges. On day 12, the lesions of both groups were in the final process of re-epithelialization, with no crust and contracted edges meeting in the center of the lesion. On day 14, the lesions had been re-epithelialized in both groups (Figure 2A).

Based on the histological images, on day 4, the vehicle group showed greater edema while the insulin group had more inflammatory cells. On day 7, the control group showed a large amount of ‘empty space’, suggesting persistent edema and lack of tissue re-organization, while the insulin group showed more advanced re-epithelialization, with less edema, more new blood vessels, more inflammatory cells, and more organized granulation tissue compared with the control group. On day 10, lesions in both groups showed a tendency towards complete re-epithelialization. However, the insulin group stood out due to the presence of more oriented and organized cells and less edema compared with the vehicle group, indicating a healing effort in the insulin group. On day 14, inflammation was absent and there was complete re-epithelialization in both groups, especially in the insulin group, which showed new epidermis and dermis (Figure 2B and C).

Topical insulin gel accelerated wound healing, showing greater re-epithelialization at day 10 (mean 64.7±12.3% for the vehicle group and 73.4±4.6% for the insulin group, T(10)=-1,778, P<0.05; n=6 per group) (Figure 2D). All hyperglycemic animals used in this study had blood glucose levels equal to or greater than 350 mg/dL with no difference in body weight and capillary blood glucose between groups (Table 1).

**Table 1:** Capillary blood glucose (CBG) and body weight changes due to streptozotocin treatment (50 mg/kg per day × 5 days, intraperitoneal).

	Vehicle group (n=44)	Insulin group (n=45)	P
CBG at DM confirmation date (mg/dL)	374.8±103.7	369.3±110.5	0.997
CBG at the time of the injury (mg/dL)	352.7±131	359±135	0.780
CBG date of excision of the injured tissue (mg/dL)	421.3±97	456.6±59	0.133
Body weight at the first dose of streptozotocin (g)	20.8±1.7	20.7±1.4	0.230
Body weight at the time of the injury (g)	20.8±2.3	20.9±2.1	0.306
Body weight at excision of the wound (g)	20.7±2.4	20.9±2.9	0.735

The data are reported as means±SD of 4-9 animals per group. DM: diabetes mellitus. Student's *t*-test.

**Figure 3:**
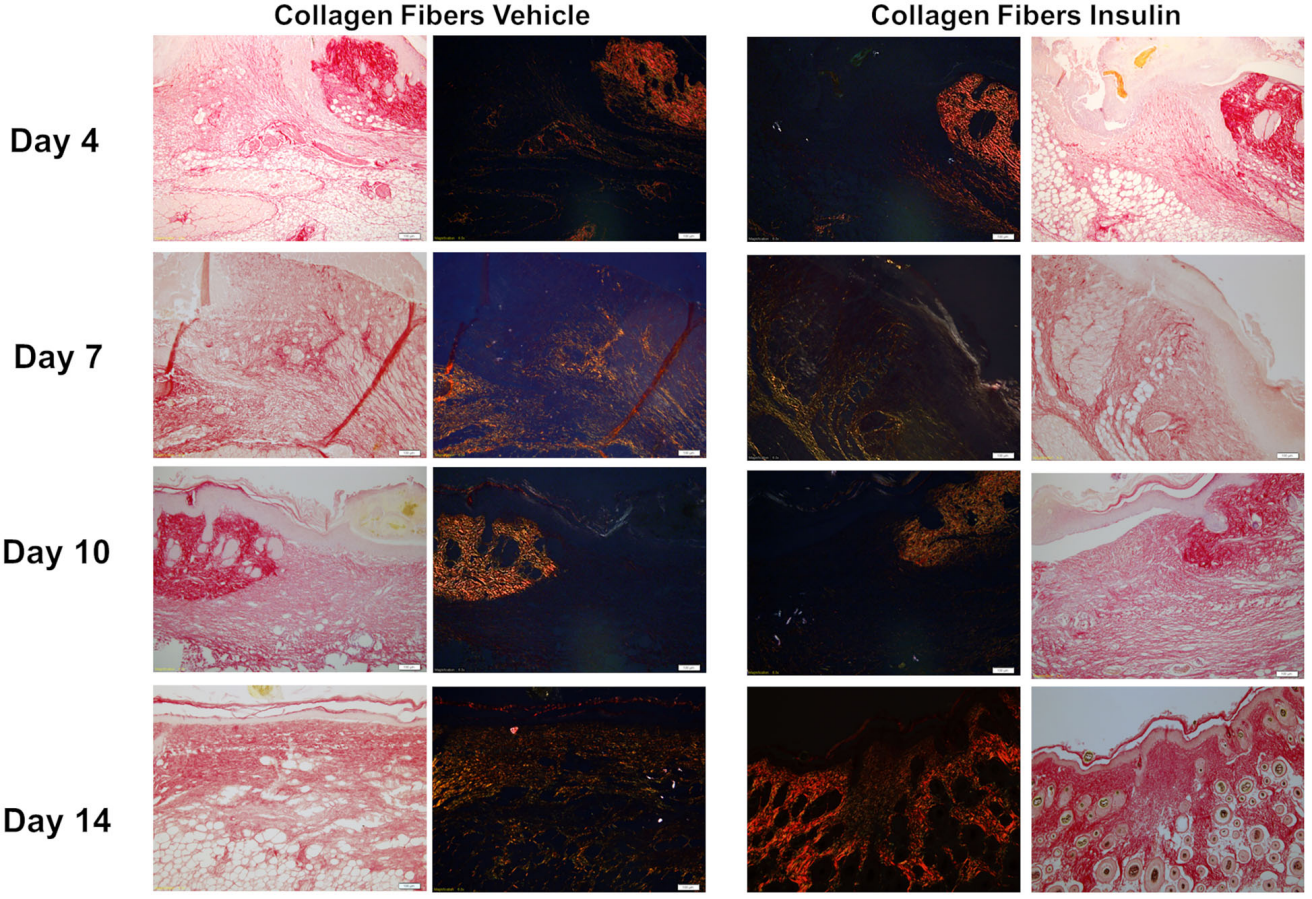
Photomicrographs of picrosirius red-stained tissue sections with and without polarized light from the vehicle and insulin groups on days 4, 7, 10, and 14 post-injury. Magnification: 10×; scale bar 100 μm.

Picrosirius red staining was performed to improve the specificity of the evaluation of collagen deposition. There were more collagen fibers in the insulin group compared with the vehicle group, with more intense, compact and reorganized staining on day 14 (Figure 3). These findings indicated that on day 14, the healing process was more advanced in the insulin group than in the vehicle group.

**Figure 4:**
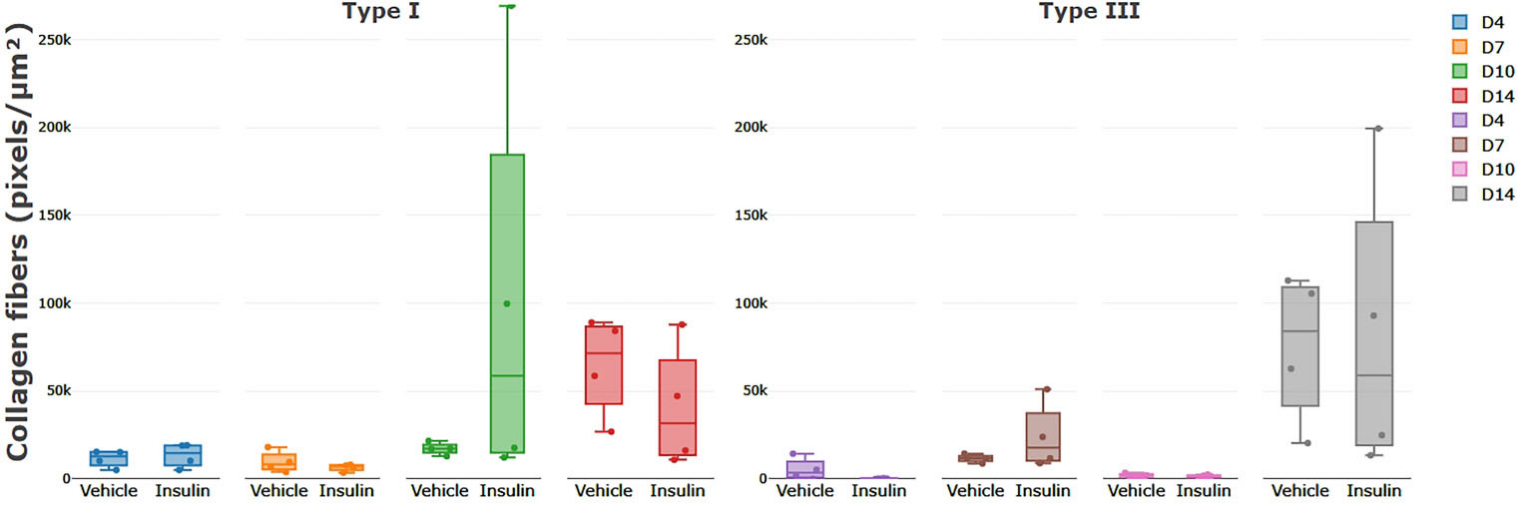
Collagen fibers in lesions of the vehicle and insulin groups on days 4, 7, 10, and 14 post-injury. Data are reported as means±SD. P>0.05, Student's *t*-test.

Regarding collagen fibers, on days 7 and 10, the insulin group had 2.05 times more type III collagen fibers than the vehicle group, but the difference was not significant (Figure 4). On day 10, the insulin group had 5.7 times more type I collagen fibers than the vehicle group, but the difference was not significant (Figure 4).

### Topical insulin gel modulated proteins of the insulin signaling pathway

**Figure 5:**
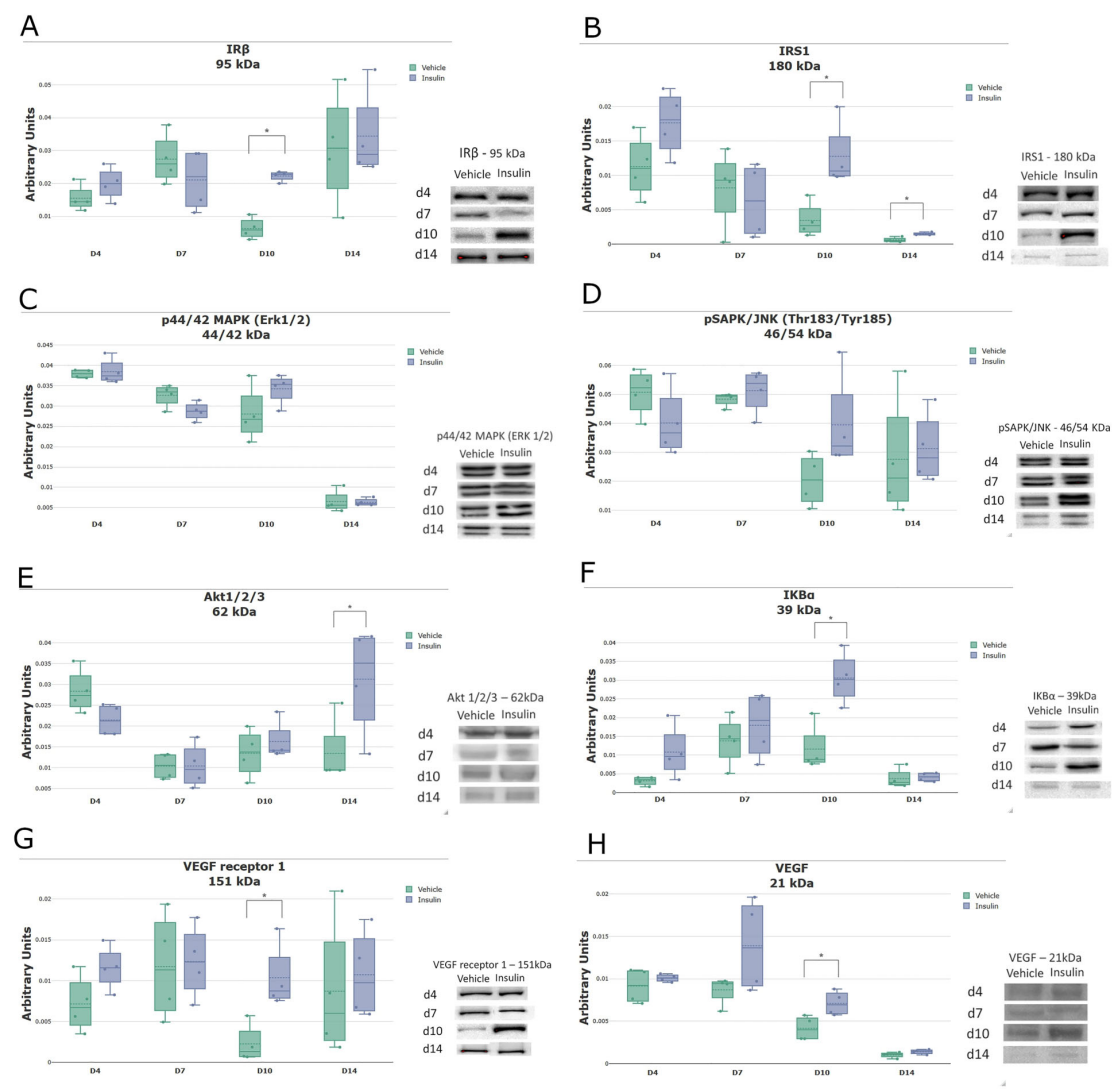
Expression of proteins in the insulin signaling pathway in lesions of the vehicle and insulin groups on days 4, 7, 10, and 14 post-injury: IRβ (**A**), IRS1 (**B**), p44/42 MAPK (Erk1/2) (**C**), pSAPK/JNK (**D**), Akt1/2/3 (**E**), IKBα (**F**), VEGF receptor 1 (**G**), and VEGF (**H**). The data (arbitrary units normalized according to the Ponceau method) are reported as medians and interquartile ranges (n=4 per group). *P<0.05, Student's *t*-test.

The expression of IRβ, IRS1, p44/42 MAPK (Erk1/2), pSAPK/JNK, Akt1/2/3 (E), IKBα, VEGF receptor 1, and VEGF (Figure 5A-H) in the vehicle and insulin groups were determined by western blotting. There were no significant differences between the groups on days 4 and 7. On day 10, there was significantly higher expression of IRβ, IKBα (both P<0.005), IRS1, Akt1/2/3, VEGF receptor 1, and VEGF (all P<0.05) in the insulin group. On day 14, there was higher expression of IRS1 and Akt1/2/3 (both P<0.05) in the insulin group. For p44/42 MAPK (Erk1/2) and pSAPK/JNK, there were no significant differences between the groups.

### Topical insulin gel modulated growth factors, cytokines, and chemokines during the healing process

**Figure 6:**
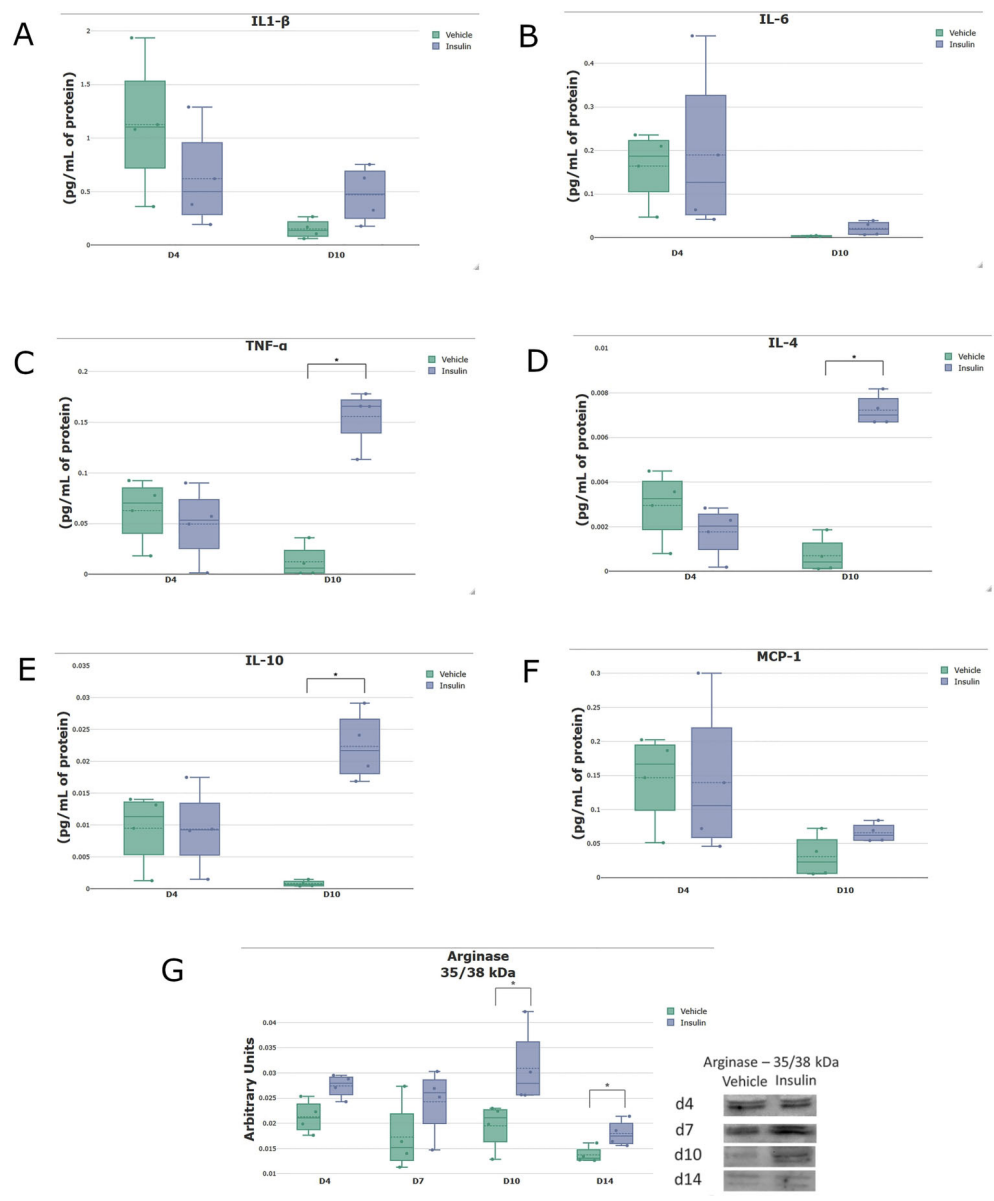
Concentration of the cytokines interleukin (IL)-1 β (**A**), IL-6 (**B**), tumor necrosis factor (TNF)-α (**C**), IL-4 (**D**), and IL-10 (**E**) and the chemokine MCP-1 (**F**) on days 4 (n=3) and 10 (n=4) post-injury in the vehicle and insulin groups. Bio-Plex immunoassays were used to measure the proteins. Tissue protein levels for arginase I (**G**) in the vehicle and insulin groups on days 4, 7, 10, and 14 post-injury (n=4 per group) using western blotting and normalization by the Ponceau method. The data are reported as medians and interquartile ranges. *P<0.05, Student's *t*-test.

**Figure 7:**
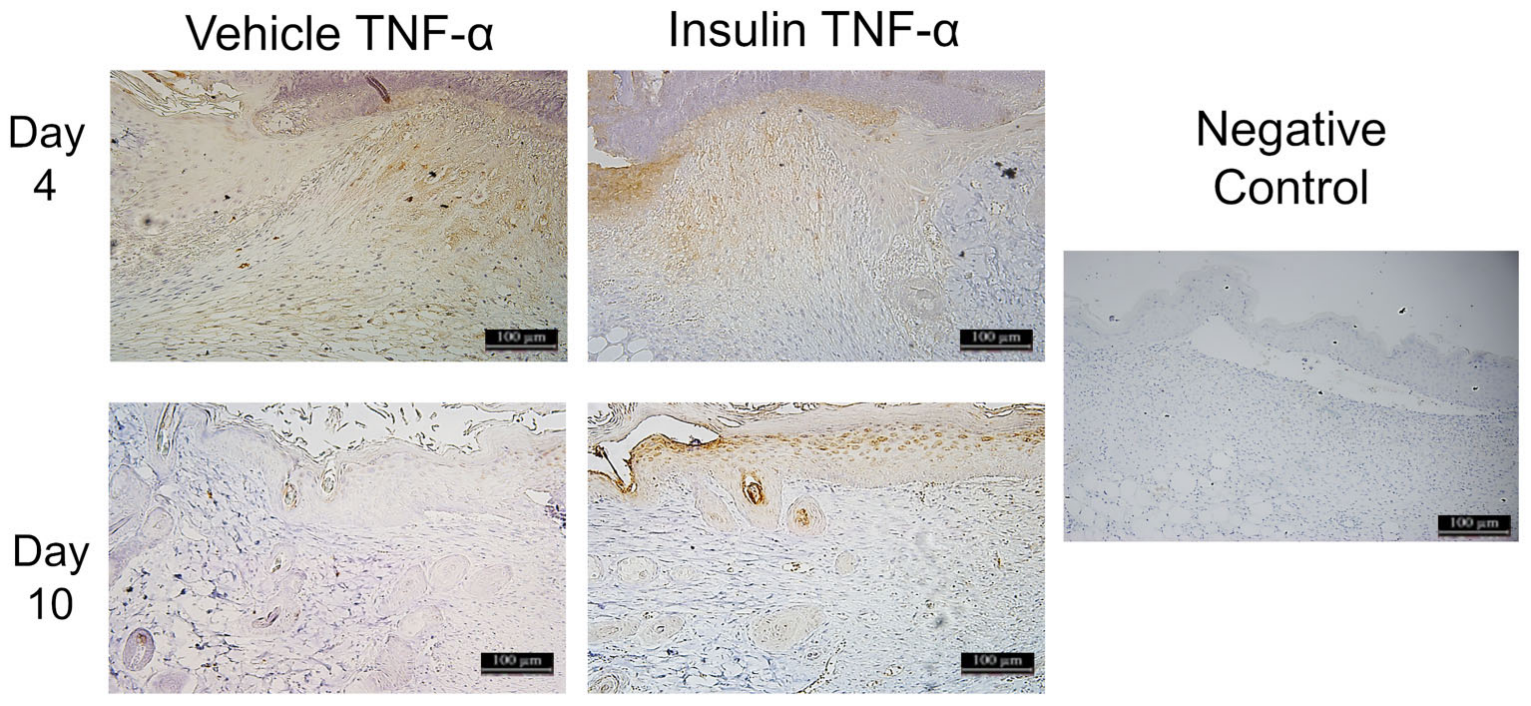
Photomicrograph of tumor necrosis factor (TNF)-α immunostaining in the wound areas for the vehicle and insulin groups on days 4 and 10 post-injury. Magnification: 10×; scale bar 100 μm. The negative control was performed without the primary antibody.

**Figure 8:**
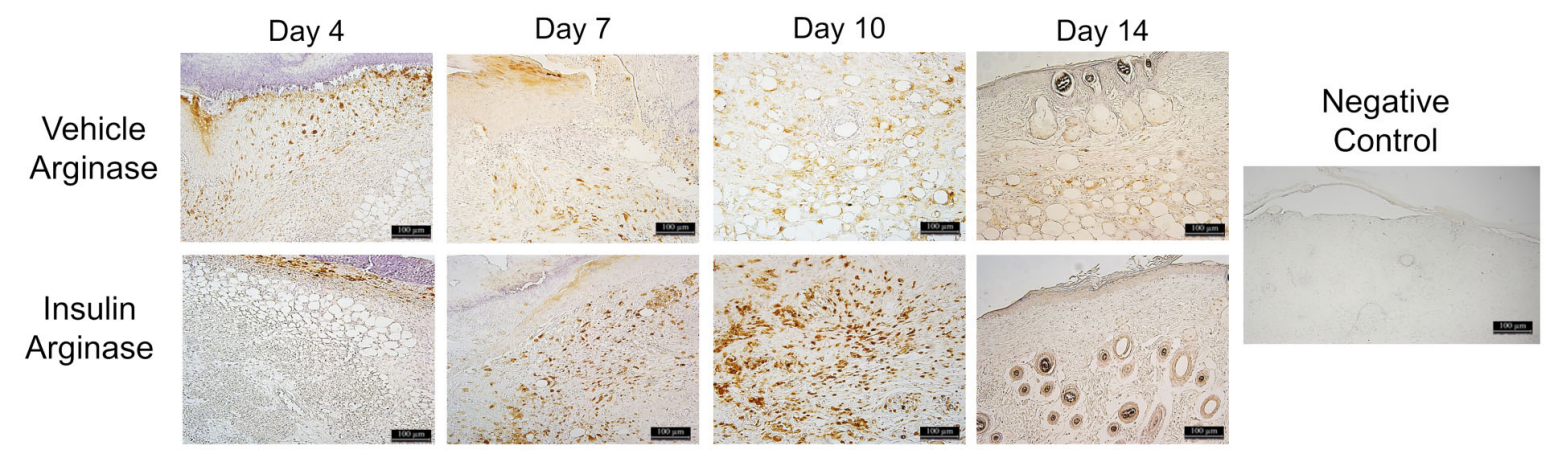
Photomicrographs of arginase I immunostaining in the wound areas for the vehicle and insulin groups on days 4, 7, 10, and 14 post-injury. Magnification: 10×; scale bar 100 μm. The negative control was performed without the primary antibody.

The levels of the pro-inflammatory cytokines IL-1β, IL-6, and TNF-α (Figure 6A-C) were analyzed in the homogenates of lesions from the vehicle and insulin groups on days 4 and 10 post-injury. There were no differences in IL-1β and IL-6 between the groups. TNF-α expression was higher in the insulin group on day 10 (P<0.005). With immunohistochemistry, it was possible to see similar TNF-α in the connective tissue of both groups on day 4. On day 10, the insulin group showed accentuated ochre staining of the epidermis, which was different from the vehicle group that did not show such staining (Figure 7). The insulin group had significantly higher expression of the anti-inflammatory cytokines IL-4 and IL-10 (Figures 6D and E) on day 10 post-injury compared with the vehicle group (P<0.005). The infiltration of macrophages was evaluated based on the expression of MCP-1 (Figure 6F) by a Bio-Plex immunoassay and arginase I (Figure 6G) by western blotting. There was great recruitment of monocytes on day 4, with a subsequent decrease on day 10, and no significant differences between the groups. The insulin group showed higher arginase I expression on days 10 and 14 (P<0.05) compared with the vehicle group. This result was confirmed by immunostaining on days 4, 7, 10, and 14 post-injury, with a noticeable increase in arginase I staining on day 10 in the insulin group compared with the vehicle group (Figure 8).

## Discussion

Delayed healing of diabetic wounds is a multifactorial, nuanced, and interconnected complication that results in significant clinical morbidity (3). Topical treatments can favor tissue repair and thus potentiate the healing of these lesions. The present study sought to characterize the potential beneficial effect of topical insulin in a gel formulation, because gel-based dressings have the ability to donate moisture to the wound bed, facilitate cell migration and proliferation, and protect granulation tissues by not adhering to the wound bed.

This study demonstrated that insulin gel increased the granulation tissue, characterized by more inflammatory cells in the first days of the healing process and more advanced healing on day 10, followed by tissue reorganization and greater deposition of type I collagen fibers on day 14. These findings corroborated the study by Schäffer et al. (17), who found that insulin treatment favors increased epithelialization, cell proliferation, and the formation of collagen fibers. Collagen production is an important regulating factor for cell migration and proliferation (18), and deficient wound healing has been associated with a reduction in collagen fibrils (19). A previous study of second-degree burns in hyperglycemic animals and treatment with topical insulin cream showed that it improves and accelerates the synthesis, deposition, and organization of collagen fibers, with an increase in synthesis of type III collagen fibers and organization of type I collagen fibers (9).

Studies have reported that topical insulin can modulate diabetic wound healing in type I and type II DM rodent models (8,20). Type I DM results from a lack of insulin secretion, not insulin resistance. Researchers have shown that topical insulin can improve wound healing by reversing the effects of inadequate insulin (8,21). Previous research has shown that topical insulin cream improves the expression of proteins involved in the insulin signaling pathway (8). Similarly, the results of this study with topical insulin gel showed positive effects on healing, with improved expression of the Irβ protein IRS1. In addition, there were no differences in the phosphorylation of pMAPK (Erk1/2) and pSAPK/JNK pathway components in the scar tissue of hyperglycemic animals between the vehicle and insulin groups at any time point. However, there was higher expression of the Akt protein with a significant difference on day 14, which decreased in the vehicle group, suggesting that insulin gel favors activation of the insulin signaling pathway via Akt. In addition, the IKK protein showed significantly higher expression in the insulin group on day 10. Moreover, the expression of VEGF receptor 1 and VEGF were higher in the insulin group on day 10, suggesting that insulin modulates angiogenesis through better expression of VEGF receptor 1, as described in the literature (19). Thus, our results suggested that the insulin signaling pathway is activated by insulin gel, with an emphasis on the Akt and IKK/NFKB pathways.

The Akt pathway is important in the activation of angiogenesis by VEGF (22). Besides, the Akt pathway plays a central role in mediating many of the insulin actions and regulates the expression and activity of a wide range of proteins, including enzymes, transcription factors, cell cycle regulatory proteins, and apoptosis and survival proteins (23,24). Activation of the Akt protein is an important step for the release of VEGF in cutaneous wounds via a post-transcriptional mechanism in keratinocytes (24-26). In addition, VEGF is necessary for vascular maturation and angiogenesis during the healing of cutaneous wounds (27). Akt can phosphorylate and activate IKK. This protein is extremely important, as it inhibits NF-κB when it is linked to it. The healing process and the NF-κB signaling pathway share a close connection at the molecular level, with NF-κB being associated with cell proliferation, adhesion, inflammation, and elimination of ROS (28). NF-κB has been implicated in the healing of corneal epithelial wounds (29) and scratches (30) and healing of skin wounds (28). The IKK kinase complex is the central element of the NF-κB cascade and, consequently, of the wound healing process.

Topical insulin can act via Akt to increase VEGF and induce the phosphorylation and activation of eNOS in the bone marrow, with consequent mobilization of endothelial progenitor cells into the circulation. SDF-1α induces these cells to the injury site, where they participate in neovasculogenesis (8). During the tissue repair process, angiogenesis plays a role in providing nutrients and oxygen to accelerate wound healing (3).

The expression of pro- and anti-inflammatory proteins was also analyzed, as these are involved in different aspects of the tissue repair process. There were no differences between the vehicle and insulin groups in the expression of the proinflammatory cytokines IL-1β and IL-6 on days 4 and 10 post-injury. On the other hand, there was a significant increase in TNF-α in the insulin group on day 10 post-injury. These data were confirmed by immunostaining, which showed similar TNF-α staining in both groups on day 4, with increased staining in the connective tissue. In contrast, TNF-α immunostaining was significantly higher on day 10 in epithelial tissue and attachments, but it was decreased in the vehicle group. Proinflammatory cytokines play a central regulatory role in the process (31) because they are among the first factors produced in response to skin damage, regulate the functions of immune cells during regeneration, and affect keratinocytes and fibroblasts (32). High expression of proinflammatory cytokines is characteristic of non-healing skin wounds in DM (3). The induction of moderate inflammation by biomaterials promotes wound healing, suggesting an important role in the balance of pro-inflammatory signals in the course of tissue regeneration (33,34). TNF-α, a monocyte-derived cytokine, is a potent inflammatory mediator that induces the production of cytokines, the activation of adhesion molecules, and the stimulation of cell growth; its effects are usually related to inflammation and cell death (35). The effects of TNF-α are dependent on its concentration and the duration of exposure, which emphasizes the importance of balancing the pro-inflammatory signals controlling wound healing. At low levels, TNF-α can promote wound healing by indirectly stimulating inflammation and increasing macrophage production of growth factors (36).

Insulin has pleiotropic roles, with important functions in cell homeostasis, through which it mediates cell and tissue regeneration, while also promoting angiogenesis through the NF-κB-dependent pathway (37). The insulin group showed higher expression of the anti-inflammatory cytokines IL-4 and IL-10 on day 10, which suggested that the insulin gel was able to switch from a pro-inflammatory to an anti-inflammatory profile. IL-10 is a regulatory cytokine with important functions in controlling inflammation during wound healing. Studies have proposed that increased IL-10 levels may induce healing without the formation of hypertrophic scars (38), reduce the inflammatory response in the wound bed, and produce an environment that is favorable for macrophage differentiation into the anti-inflammatory M2 profile (39,40). Moreover, IL-4 promotes the polarization of macrophages from the pro-inflammatory to the anti-inflammatory profile. Macrophages play an essential role in the different phases of tissue repair, with participation in both the destructive and repair functions of the healing process (40).

Macrophage plasticity associated with elevation of anti-inflammatory cytokines in the present study led to the investigation of macrophage markers. MCP-1 level was similar in both groups on days 4 and 10 post-injury. Moreover, considering macrophage marking, the expression of arginase I was quantified by western blotting and immunostaining. On days 10 and 14 post-injury, there was a higher arginase 1 expression in the insulin group. Arginase 1 is classically considered to be a marker of M2 macrophages, which are alternatively activated by IL-4 and have an important function in the metabolic pathway of arginine, where they direct arginine metabolism into arginase with the consequent promotion of tissue repair by collagen synthesis. These arginase 1 results correlated with the findings of a greater quantity and organization of type I and III collagen fibers assessed by Sirius red staining. In this context, topical insulin gel might represent a promising therapeutic agent given that it modulated the anti-inflammatory cytokines IL-4 and IL-10, with improvements in the expression of arginase I and type 1 collagen fibers. In addition, it promoted increased VEGF and VEGF receptor 1 and recovery in the expression of proteins in the insulin signaling pathway.

Several limitations of this study are worth noting. Although arginase 1 is a good M2 macrophage marker, it can also be produced by fibroblasts, which could bias the results. Glucose levels and ROS production were not quantified in the tissue homogenates to determine whether the effect of topical insulin was direct or indirect. There was not a complete analysis of the insulin pathway of phosphorylated proteins. Finally, only male mice were used. Additional studies with female mice are required.

In the present study, topical insulin gel effectively promoted the re-epithelialization process *in vivo*; regulation of the insulin signaling pathway, pro-inflammatory proteins, and VEGF/VEGF receptor 1 may be the mechanism of action. Topical insulin gel activated downstream pathways and enhanced Akt and VEGF, with the upregulation of NF-κB. There was also different expression of the anti-inflammatory cytokines IL-4 and IL-10, associated with the modulation of arginase 1 (an M2 macrophage marker). The expression of other autophagy-related proteins in preclinical studies in wound healing needs further investigation to substantiate the current findings.
